# Dr. Pierce J. O’Brien

**Published:** 1912-07

**Authors:** 


DR. PIERCE J. O'BRIEN of Troy, died May 26, aged 38. He was a graduate of Albany, 1898.

